# Modeling Cardiomyopathies in a Dish: State-of-the-Art and Novel Perspectives on hiPSC-Derived Cardiomyocytes Maturation

**DOI:** 10.3390/biology10080730

**Published:** 2021-07-30

**Authors:** Francesco Lodola, Verónica Celeste De Giusti, Claudia Maniezzi, Daniele Martone, Ilaria Stadiotti, Elena Sommariva, Angela Serena Maione

**Affiliations:** 1 Department of Biotechnology and Biosciences, University of Milano-Bicocca, Piazza della Scienza, 2, Ed U3, 20126 Milan, Italy; claudia.maniezzi@unimib.it (C.M.); daniele.martone@unimib.it (D.M.); 2 Centro de Investigaciones Cardiovasculares, Facultad de Ciencias Médicas, Universidad Nacional de La Plata-CONICET, La Plata 1900, Argentina; degiustiveronica@gmail.com; 3 Vascular Biology and Regenerative Medicine Unit, Centro Cardiologico Monzino IRCCS, 20138 Milan, Italy; ilaria.stadiotti@ccfm.it (I.S.); elena.sommariva@ccfm.it (E.S.)

**Keywords:** hiPSC-CMs, cardiomyopathies, cardiovascular disease modeling, patient-specific medicine, stem cell maturation

## Abstract

**Simple Summary:**

Cardiomyopathies modeling is greatly smoothened by the technological advances made in the use of human induced pluripotent stem cells derived cardiomyocytes (hiPSC-CMs). Despite the advantages of allowing to model patient specific disease, hiPSC–CMs still show a degree of maturity comparable to fetal CMs. In this perspective, we discuss different methods to improve hiPSC-CMs maturity, and to create cardiomyopathy-specific models, allowing the assessment of relevant phenotypes. In addition, current limitations and required evolutions in cardiomyopathy disease modeling are addressed.

**Abstract:**

The stem cell technology and the induced pluripotent stem cells (iPSCs) production represent an excellent alternative tool to study cardiomyopathies, which overcome the limitations associated with primary cardiomyocytes (CMs) access and manipulation. CMs from human iPSCs (hiPSC–CMs) are genetically identical to patient primary cells of origin, with the main electrophysiological and mechanical features of CMs. The key issue to be solved is to achieve a degree of structural and functional maturity typical of adult CMs. In this perspective, we will focus on the main differences between fetal-like hiPSC-CMs and adult CMs. A viewpoint is given on the different approaches used to improve hiPSC-CMs maturity, spanning from long-term culture to complex engineered heart tissue. Further, we outline limitations and future developments needed in cardiomyopathy disease modeling.

## 1. Introduction

Cardiomyopathies are inherited cardiac conditions basically classified as myocardial disorders in which the heart muscle is structurally and functionally abnormal in the absence of ischaemic heart disease [1]. Therefore, the inherited cardiomyopathies are usually classified based on morphological alterations or electrical alterations. In particular, structural cardiomyopathies have historically been classified according to phenotypic features in different main categories: hypertrophic, dilated, arrhythmogenic, and restrictive [1,2]. Channelopathies are likewise grouped as arrhythmic disorders and ascribed to altered ion channel function or intracellular calcium handling disorder, causing electrical instability, and leading to malignant arrhythmias [3,4,5]. The main cardiac channelopathies are long QT syndrome (LQTS), short QT syndrome (SQTS), Brugada syndrome (BrS), and catecholaminergic polymorphic ventricular tachycardia (CPVT).

An adult human heart is made up of different cell types essential for proper cardiac function. The most substantial volume is covered by cardiomyocytes (CMs) surrounded by highly specialized cells like fibroblasts, endothelial cells, and vascular cells [6,7,8]. Non-myocyte cells are crucial for tissue homeostasis and cell-cell interaction based on their role in maintaining the structure, mechanical and electrical heart functions as occurs for fibroblasts and mesenchymal stromal cells [9,10,11].

The use of primary CMs for biochemical experiments is mostly limited by the cell number that can be isolated from mammalian species and by the restricted ability for CM division in culture and have a limited lifetime *in vitro*. In addition, cardiac biopsies are rarely available as a source for CMs. The limitations associated with adult CM manipulation have been balanced by stem cell technology and the induced pluripotent stem cells (iPSCs) production. They represent a useful and versatile tool to study cardiomyopathies based on their ability to differentiate in several cell types, including cardiac ones that are genetically identical to the cells of origin. Methods for controlling hiPSCs reprogramming and differentiation have been greatly improved over the past decade [12,13]; in particular, great efforts have been made to improve the maturity of iPSC-derived CMs (iPSC-CMs) which represent a major asset for cardiovascular diseases modeling and for personalized and precision medicine [14].

The iPSCs features make them highly suitable for generating “disease-in-a-dish” models: (i) the genetic profile of each iPSC line is analogous to founder cells; (ii) iPSCs allow the study of the genetic contribution to the disease phenotypes; (iii) by using genome-editing tools, such as CRISPR/Cas9 technology, it is possible to modify the genetic profile of iPSCs. The DNA sequence can be modified either by introducing a specific pathological mutation into control iPSC lines or by correcting a causative mutation [15].

*In vitro* single-cell models have been so far essential to the study of the pathogenic mechanisms underlying cardiomyopathies, but there are several limitations to their use. Isolated cells lack some important features of *in vivo* systems such as homo- and hetero-cellular interactions that are crucial for electrical properties contributing to the propagation of electrical impulses or for mechanical properties, creating a functional syncytium. Multicellular interactions *in vivo* may result in a worsening of a pathological phenotype that might not be evident in a single cell model. The implementation of a multicellular model can better recapitulate the complexity of the cardiomyopathies. The reciprocal interactions between myocyte and non-myocyte cell types in cardiomyopathies represent the first step towards personalized “heart in a dish” to model these diseases. From this perspective, we will focus the attention on hiPSC-CMs, we will critically discuss their current limitations and approaches to improve their maturation.

## 2. Structural and Functional Features of hiPSC-Derived CMs

hiPSC cardiomyogenic differentiation protocols have been developed in order to use hiPSC-CMs as a tool to study the basic mechanisms of human inherited cardiomyopathies. hiPSC-CMs exhibit heterogeneous phenotypes with diverse and specific characteristics depending on the differentiation protocol. A compelling issue that limits hiPSC-CMs bench-to-bedside translation is whether in vitro hiPSC-CMs culture is actually able to recapitulate key-enabling electrophysiological and mechanical properties of mature cardiomyocytes. In the next paragraphs, we will discuss the principal structural, electrophysiological, Ca^2+^ handling, and bioenergetics properties of hiPSC-CMs in comparison to adult myocytes.

### 2.1. Morphological Characteristics

**Figure 1:**
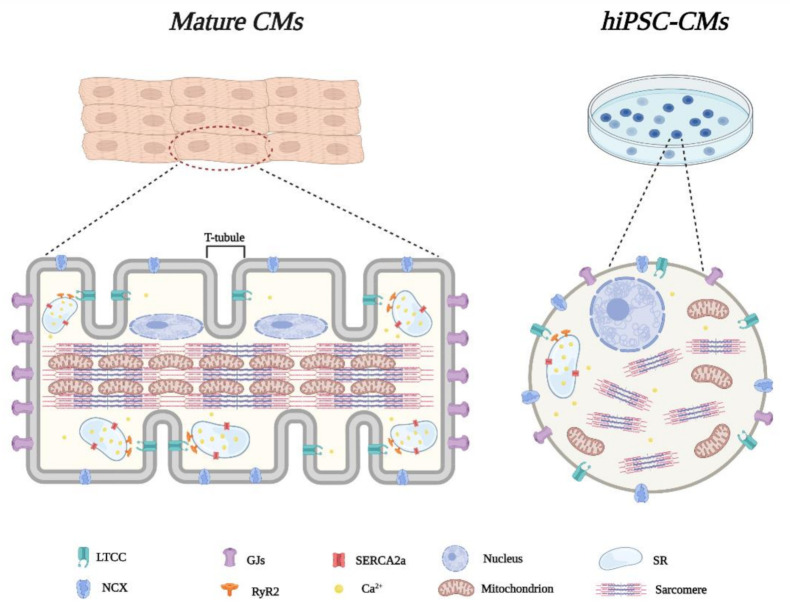
Principal morphological characteristics of the two cell categories: on the left a mature cardiomyocyte (CM) with a typical rod-shaped structure and specific organization, an elaborate tubular network, a neighboring sarcoplasmic reticulum (SR), and large numbers of mitochondria. On the right, the human-induced pluripotent stem cells (hiPSC)-CM, which is smaller and more round-shaped, with an immature structure. Compared to adult CM, the hiPSC-CM presents (i) a lower co-localization of the L-type Ca^2+^ channel (LTCC) with the Ryanodine receptor type 2 (RyR2), (ii) a reduced number of gap junctions (GJs), (iii) an irregular distribution of Ca^2+^ ions and (iv) the T-tubular structure deficiency and immature sarcomere organization.

Morphology is an important aspect of the phenotype of a myocyte since it not only defines the structural scenario, but is also critical to determinate the cell’s electrical and contractile properties. Adult CMs are typically rod-shaped with an average size of 100–150 μm in length and 15–30 μm in diameter [16]. They are highly organized and present a series of unique and defined structures that are vital for the proper functioning of the heart (Figure 1); the elaborate transverse-tubule (T-tubule) network and neighboring sarcoplasmic reticulum (SR) is critical for beat-to-beat heart contraction while the large numbers of mitochondria make them highly fatigue-resistant [17]. Force development is strongly dependent on sarcomere content and myofibril distribution. The former is the repeating unit of the latter and constitutes the myocyte fundamental building block, responsible to convert the chemical energy of adenosine triphosphate (ATP) into mechanical work. Importantly, although CMs are individual units anatomically, they function as a syncytium and are coupled via polarized intercalated disc complexes, which allow action potential propagation with a propagation speed of ~100 cm/s [18].

By contrast, as depicted in Figure 1, conventional hiPSC-CMs are smaller (5–10 μm in diameter), more round-shaped than the adult myocytes, and exhibit an immature irregular structure [19]. In particular, they lack an organized T-tubular network resulting in a poor colocalization of the L-type calcium channel Cav1.2 and Ryanodine receptor 2 (RyR2) [20]. This leads to an unequal distribution of Ca^2+^ release in the cytoplasm with global Ca^2+^ signals substantially slower than in adult CMs [20]. Furthermore, the gap junctions are less expressed and circumferentially distributed; therefore, the electrical conduction speed is an order of magnitude slower (~10 cm/s) than in adult cells. [21]. Sarcomere/T tubule irregularity also induces a disorganized contraction [22]. Moreover, hiPSC-CMs present less abundant mitochondria, localized mainly at the perinuclear space (occupying less than 5% of total cell volume) [21,23].

Altogether, these characteristics, that resemble those of rudimentary myocytes, lead to functional variances that could limit the potential use of hiPSC-CMs in disease modeling and therapeutic regeneration strategies.

### 2.2. Electrophysiological Properties

The shape of the cardiac action potential is characterized by the different expression levels and functionality of voltage-gated ion channels, consistent with the ion currents across the plasma membrane of the myocytes [24]. Despite the discrepancies seen, some electrophysiological traits are common in hiPSC-CMs obtained with standard differentiation protocols. These cells present a maximum diastolic potential more depolarized (~−60 mV) than adult CMs (~−80 mV), mainly caused by a reduced outward flux of potassium correlated to low levels of inward rectifier current (I_K1_) [25]. Furthermore, the decreased I_K1_, in conjunction with altered kinetics of the sodium channel Nav1.5, pours into a slower upstroke velocity (10–60 V/s) compared to the human native counterpart, normally close to 200–300 V/s. Unlike the latter, the hiPSC-CMs exhibit also autorhythmicity; this is due to the higher levels of the pacemaker current I_f_ (encoded by HCN4) that synergically interacts with the “Ca^2+^ clock” mechanism causing spontaneous activity in a similar way to what happens in sinoatrial nodal cells [26,27]. Other phenotypical hallmarks of hiPSC-CMs action potential waveform are: (i) a less prominent transient repolarization period (i.e., the notch, phase 1), attributable to a reduced transient outward potassium current [28]; (ii) anticipated repolarization due to the lack of the plateau phase (phase 2) and a fast phase 3, explicable with an altered balance between the inward currents, mediated by the L-type Ca^2+^ channels and the sodium–calcium exchanger (I_NCX_), and the outward currents, regulated by the rapid (I_Kr_) and slow (I_Ks_) delayed-rectifier potassium currents [29]. Nevertheless, differentiation methods have a major impact on hiPSC-CMs functional characteristics. Recently, groundbreaking approaches, which will be described in more detail later, allowed to obtain more physiological upstroke velocities and ventricular-like cells [30,31,32], thus further suggesting the compelling need to optimize differentiation protocols to obtain cells electrically closer to adult CMs.

### 2.3. Excitation-Contraction Coupling and Ca^2+^ Handling

The term excitation-contraction (EC) coupling describes the sequence of events that link CM excitation to contraction via intracellular Ca^2+^ release from the sarcoplasmic reticulum (SR) [33]. Briefly, in adult cardiac cells the action potential propagation along the transverse-axial tubular system leads to L-type Ca^2+^ channels opening on the plasma membrane, allowing Ca^2+^ entry from the extracellular space. This phenomenon triggers a further massive Ca^2+^ spill into the cytoplasm from the RyR2 on the SR, a phenomenon called Ca^2+^-induced Ca^2+^-release (CICR). The subsequent binding of cytoplasmic Ca^2+^ to the sarcomeric protein troponin C causes the conformational shift in tropomyosin, which ultimately translates to myofilament sliding and contraction. For relaxation to occur, Ca^2+^ must be removed from the cytoplasm, partly pumped back to the SR via the sarcoplasmic/endoplasmic reticulum Ca^2+^ ATPase 2a (SERCA2a) or out of the cell via the sodium–calcium exchanger (NCX). For the sake of completeness, the mitochondrial Ca^2+^ uniporter (MCU) and the sarcolemmal Ca^2+^ ATPase (PMCA) are also involved, but they play a minor role in the re-equilibration process [34].

The efficiency of these processes is strictly correlated not only to the expression of specific Ca^2+^-handling proteins but also to the specific spatial organization of the Ca^2+^ machinery [33]. In hiPSC-CMs, even if with different expression patterns, it has been observed the presence of functional EC coupling; however, the immature ultrastructural conformation (i.e., lack of T-tubules, neonatal-like sarcomere organization) strongly impairs its functionality causing strong repercussions on Ca^2+^ dynamics [20]. Moreover, contractile force is strictly dependent on a well-structured sarcomere network. The smaller length and the poor localization and alignment likely contribute to the overall lower force seen in hiPSC-CMs cells compared to adult ventricular CMs [20].

### 2.4. Metabolism

Cardiac metabolism dramatically changes during maturation. In adult myocytes, mitochondria cover one-third of the cell volume and are well organized in functional complexes among SR and myofibrils. The majority (~80%) of total energy consumption is supplied by β-oxidation of fatty acids and the ATP produced is used almost entirely to support contractile function and activity of transport ATPases. hiPSC-CMs behave more like immature myocytes with their metabolic consumption mainly relying on glycolysis which is relatively inefficient from an energetic standpoint [23].

## 3. hiPSC-CMs State-of-the-Art and Novel Approaches for CMs Maturation

The concept of “CM maturation” refers to the body of changes affecting cell structure, metabolism, functionality, and gene expression that happen during the transition from fetal to adult developmental state. In the next paragraph, we will discuss consolidated approaches and cutting-edge methodologies to pursue further maturation of hiPSC-CMs.

### 3.1. Long-Term Culture and In Vivo Maturation

The analysis of hiPSC-CMs obtained after different times of cardiac differentiation revealed increasing readouts of maturity in the function of the time and stimuli. However, even with long term culture maturation [35,36], hiPSC-CMs still retains some features of the embryonic phenotype such as the absence of a mature sarcomeric structure with M-bands and a variable degree of myofibrillar organization, not comparable to the degree of maturity reached by transplanting them in rat neonatal hearts [37].

To date, the long-term 2D culture condition has been assessed up to one year of cardiac differentiation in order to test the hiPSC-CMs maturation enhancement [38,39]. Prolonged culture leads to a more mature phenotype of hiPSC-CMs in terms of morphology (larger cells), structure (visible sarcomeric organization), physiology (Ca^2+^ handling properties), and electrophysiological features (increased action potential amplitudes) [39]. Notably, at 180 days of culture iPSC-CMs show more tightly packed myofibrils, and mature Z, A, H, and I bands are visible. In contrast, the M bands that are essential in the sarcomere structure develop after 1 year of culture confirming that prolonged culture generates more mature CMs [38].

Considerable maturation of hiPSC-CMs was also observed after *in vivo* cell transplantation in several adult animals like mice, rats, and non-human primates [40,41,42]. After 3 months of transplantation, hiPSC-CMs were smaller than host cells but developed partially mature sarcomeric structures. The *in vivo* induction of hiPSC-CMs maturation seems to be greater in adult rat hearts than in neonatal rat hearts. However, this difference could also be ascribed to a different cell dose used in the two experiments [43]. The improvement of *in vivo* maturation rate also occurs for pathological phenotypes as proven for the arrhythmogenic right ventricular cardiomyopathy (ARVC) hiPSC-CMs engraftment. These hiPSC-CMs, transplanted into rat hearts, are highly analogous to adult ARVC CMs in terms of morphology, Ca^2+^ transients, contractility, and gene expression [37]. Thus, when subjected to the *in vivo* microenvironment, hiPSC-CMs are more mature than *in vitro* ones. This observation implies that by *in vitro* mimicking of *in vivo* conditions it should be possible to achieve improved maturation.

### 3.2. Biochemical Cues

Several studies have highlighted the importance of controlling metabolism to generate more mature stem cell models of human cardiovascular disease. Even if the regulators and the pathways involved have not yet been fully elucidated, there is strong evidence that this approach leads to gene expression patterns and functional properties more comparable to those of adult CMs. For example, treatment with the thyroid hormone tri-iodothyronine (T3), a hormone pivotal for optimal heart growth, drives structural and functional hiPSC-CMs maturation, enhancing the size and sarcomere length as well as contractility capability, rates of Ca^2+^ release and re-uptake, and energy metabolism [44]. A further degree of maturation with functional T-Tubule development has been obtained by combining T3 with glucocorticoid hormones [45]. Rupert and Coulombe demonstrated that insulin-like growth factor-1 (IGF1), in synergy with neuregulin-1 (NRG1), enhanced proliferation, metabolic maturity, and the force-frequency relationship in hESC-Derived Engineered Cardiac Tissues [46]. A fundamental postnatal CM maturation step is the metabolic switch from glycolysis to fatty acid oxidation. Different studies have correlated culture medium properties and functional maturation of hiPSC-CMs. Indeed, several groups demonstrated that fatty acids supplementation improved hiPSC-CMs overall maturation [47,48,49], while a high-glucose media sorted the opposite effect. Kim and colleagues correlated this effect to the activation of the key metabolic regulator hypoxia-inducible factor 1α (HIF-1α) and the consequent upregulation of lactate dehydrogenase A (LDHA) [50]. This evidence was corroborated by a more recent study that demonstrated how siRNA inhibition of HIF-1α signaling shifted the cell metabolism towards glycolysis, thus highlighting how HIF-1α–LDHA is a crucial pathway for cardiac maturation [51].

### 3.3. Biophysical Stimuli

Biophysical cues are an important tool to promote the maturation of hiPSC-CMs *in vitro*. Tuning substrate properties (i.e., stiffness, topography, and conductivity) has been widely exploited to modify cell behavior [52] mimicking the *in vivo* environment. Artificial polymers like polydimethylsiloxane (PDMS) have been used to create *in vitro* scaffolds that recapitulate the extracellular matrix (ECM) [53,54,55]. Lyra-Leite and colleagues’ study indicate how mitochondrial architecture is dependent on cell shape and alignment and only moderately on matrix rigidity [56]. In agreement, the possibility to pursue a geometry-driven organization, forcing cell growth on islands of different shapes or with micro- and nano-patterned substrates, allowed regulating the structure and function of hiPSC-CMs [57,58,59,60]. In addition, electrical and mechanical stimuli have been applied to hiPSC-CMs sorting positive effects both on the structural and electrical degree of maturation [61,62,63]. Of recent interest, Dwenger et al. proposed optogenetics, a widely used biological technique that uses light to modulate molecular events through a genetically encoded component, to perform non-contact chronic optical stimulation in engineered cardiac tissues from hiPSC-CMs [64]. In comparison to the above-mentioned physical triggers, the use of light will represent a possible novel tool of modulation that offers unprecedented advantages, allowing to obtain a higher temporal and spatial selectivity net of lower invasiveness.

### 3.4. Co-Culture and 3D Cultures

Taken together, this information highlights the critical role of the microenvironment in increasing the phenotypic hallmarks of CM maturation that are lost within *in vitro* culture [65].

Moreover, the CMs fraction in the fetal heart gradually decreases with adult heart development due to the increased proliferation of non-CMs. *In vitro* data of 2D CMs and non-CMs co-culture indicate that the latest regulate CM maturation [66]. This regulation is possibly based on both direct cell-cell contact and on the paracrine factors secreted by non-CMs, modulating the CMs phenotype [67].

Cardiac fibroblasts and endothelial cells, for instance, can mimic biophysical and biochemical cues as a result of their ability to create the ECM and angiogenesis, respectively [68,69]. Nevertheless, 2D cultures cannot fully recapitulate the microenvironment, three-dimensional alignment, and the complex architectural network that occurs in an *in vivo* system. For instance, hiPSC-CMs in 3D culture develop greater structural, functional, and metabolic maturation than those in 2D culture [30,70,71,72] such as enhanced myofibrillar alignment and sarcolemma remodeling that lead to improved Ca^2+^ handling [73].

To date, different methods have been used to generate 3D cardiac cultures. The cardiac microtissues can be made by aggregation of hiPSC-CMs only or in combination with non-CMs in defined ratios through non-adhesive U-shaped wells, hanging drops, or agitation culture. Building of the 3D cardiac structure essentially involves two methods: scaffold-free, in which cells are seeded in culture medium, or scaffold-based, in which cells are seeded on a medium enriched in ECM/hydrogels or highly viscous chemicals and more complex engineering methods. By integrating tissue engineering and iPSC technology, it is possible to develop cardiac micro-physiological systems. Some examples of engineered heart tissues (EHT) include the use of biodegradable scaffolds for the generation of 3D microchannels coated with endothelial cells [74], the multiphoton-excited 3D printing technique to generate matrix scaffold with submicron resolution [75], and engineered cardiac patch with electronic properties [76].

A further approach to enhance maturation involves the formation of organoids, which provide a significant number of properties analogous to native heart tissue [77,78]. The combination of CMs and non-cardiac cells leads to the production of an aligned syncytium with tight cell-cell and cell-ECM connections characterized by a higher level of connexin-43 and improved electromechanical signal conduction [32]. In addition, the presence of matrix-secreting fibroblasts, endothelial cells, and muscle cells generates improved physiological stiffness and microvasculature within the 3D cardiac structures [79].

## 4. Modeling Cardiomyopathies *In Vitro*

Modeling cardiomyopathies *in vitro* is a challenge that is being faced by many investigators. Indeed, different issues make it difficult to address.

All cardiomyopathies show an undoubted genetic component, which is, however, incompletely understood, due to variable expressivity, incomplete penetrance, and genetic pleiotropy. In addition, a further complication comes from the existing phenotypic, and, in some cases, an underlying pathophysiological mechanism also overlaps different cardiomyopathies.

The development of hiPSC technology has provided the possibility to investigate the genetic aspects of cardiomyopathies in a patient- and mutation-specific scenario.

However, few reports revealed that epigenetic traits complicate the interpretation of iPSC-derived cell genetic-dependent readouts. Indeed, low passage iPSC retain residual DNA methylation modifications typical of their somatic tissue of origin, which influence their differentiation capabilities [80,81,82].

Given the electric etiology of the disorders, for channelopathy modeling, the main cell components are constituted by CMs [83,84,85]. Differently, structural cardiomyopathy modeling complex architecture requires the presence of several cell types. Indeed, over time, it assisted in disease modeling of increasing complexities, from the choice of the components to their patterning [86,87]. First of all, CM obtained from iPSC show an elevated degree of heterogeneity, spanning from atrial-like to nodal-like to ventricular-like [88]. Since cardiomyopathies mainly involve the ventricles, enrichment for ventricular CM may increase the proximity to the disease [89]. Further, to mimic ventricular tissue composition, researchers must aim to recapitulate the cell types, the correct amount, and distribution of adult ventricles [90]. An elaborate network of extracellular material, the ECM, either directly secreted by the cells present in the culture or provided as a support/anchoring scaffold, is also increasing the complexity of cardiomyopathy modeling, mimicking the mechanical, alignment, and chemical cues of the diseased microenvironment [91].

Different readouts are to be tested in different forms of cardiomyopathies: (i) electrophysiology and fields potentials can be monitored both in channelopathies and in structural cardiomyopathies and are essential to model arrhythmic dysfunction [92]; (ii) marker for altered pathways are readouts needed to understand the pathogenic mechanisms in every form of cardiomyopathies [93,94]; (iii) testing calcium handling dysregulation is important in channelopathies, as CPVT, but also in structural cardiomyopathies as Dilated Cardiomyopathy (DCM) and ARVC [95,96]; (iv) motion analysis and force measurements need to be tested mainly in the structural cardiomyopathies [97]; (v) similarly, the evaluation of ECM deposition is a common feature of pro-fibrotic tissue remodeling [69].

Other readouts are instead specific for certain cardiomyopathies, as occurs for fat accumulation in ARVC [98], or CM hypertrophy in HCM [99].

In addition, some cardiomyopathies (e.g., ARVC or CPVT) have a higher burden of arrhythmias and/or structural deterioration in the athletes. The effect of exercise can be modeled by mechanical stimuli or by pacing at increased frequencies [100].

**Figure 2:**
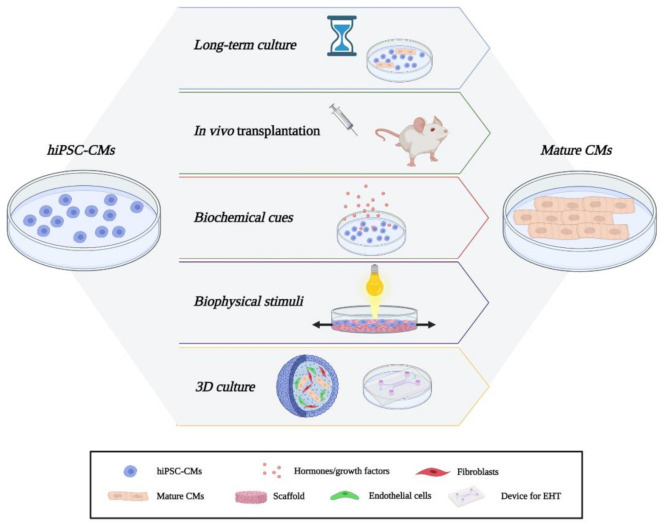
Overview of approaches to improve hiPSC-CMs maturation. Starting from the top: long-term culture condition; *in vivo* cell transplantation; biochemical induction by hormones/growth factors; biophysical modulation as occur for biomechanical and optogenetic stimuli, 3D multicellular structure generation, and EHT.

However, iPSC-CMs modeling potential is hindered due to their relative immaturity at a structural, electrical, and metabolic level in comparison to adult human CMs (Figure 1). From this perspective, we have discussed cutting-edge approaches to ameliorate the hiPSC-CMs degree of maturation so as to obtain a more reliable and accurate tool for disease modeling of cardiomyopathies (Figure 2). In particular, hiPSC-CMs maturation can be improved to a near-adult state by long-term culture [38]. Although the result is a highly mature cell in morphology and structure, its application to model cardiomyopathies is still hampered by the long lead time required for their production. The maturity reached by *in vivo* transplantation offers an overview of all the factors and environments that contribute to CM maturation (e.g., different cell type interaction, paracrine factors, biophysical stimuli) [40] that need to be recapitulated *in vitro*. Indeed, the use of biochemical and physical stimuli *in vitro* represents a tool with enormous potential. Inter alia, given the promising results obtained with optogenetics, it is conceivable that other geneless photomodulation approaches will gain momentum in forthcoming years. Examples may include photothermal stimulation [101], photoacoustic modulation [102], activation by electromagnetic field [103,104], and use of exogenous light active materials [105,106] as transducers of stimuli. In perspective, we believe that the latter seems particularly suitable to the scope thanks to the fact that they can be easily micro- and nano-structured through reasonably fast, repeatable and simple techniques [107,108,109], are highly biocompatible, and have proven capable of triggering pivotal biological targets (i.e., Ca^2+^ dynamics, ROS production) with unprecedented selectivity and spatial resolution [110,111,112,113]. Finally, co-cultures and 3D cultures provide an excellent resource to investigate myocytes and non-myocytes features and how their interplay may impact the disease phenotypes [32]. Both direct interactions and paracrine factors, as well as the contribution of ECM and/or scaffolds to shape the microtissues, can be determined to improve the differentiation protocols [114].

In conclusion, we strongly believe that the advent of new methodologies, corroborated by the synergic combination of different expertise (i.e., biochemical, physical, chemical, material science), will allow obtaining hiPSC-CMs that will fully resemble the adult CMs phenotype, thus strengthening the applicability of the technology for the development of patient-specific therapies. The main achievement of *in vitro* cardiomyopathy modeling is thus to obtain more and more complex constructs, starting from genetic components and reproducing both the structure of the myocardium and its function and external stimuli while controlling modifiable factors to reduce the biological complexity of the system.

The necessity to improve reproducibility and scalability of cardiomyopathy *in vitro* models is still an unresolved clinical need to reduce the burden of these diseases. Cardiomyopathy high throughput models will become the main form of preclinical research and will bridge the gap towards clinical trials while reducing the tests on animal models.

## Data Availability

The data presented in this study are available on reasonable request from the corresponding author. The data are not publicly available due to privacy reasons.
